# The prognostic value of NRF2 in solid tumor patients: a meta-analysis

**DOI:** 10.18632/oncotarget.19838

**Published:** 2017-08-03

**Authors:** Lingling Wang, Chunze Zhang, Litao Qin, Jingyue Xu, Xiaobo Li, Wenhong Wang, Lingqin Kong, Taizhen Zhou, Xichuan Li

**Affiliations:** ^1^ School of Basic Medical Sciences, Tianjin Medical University, Tianjin, China; ^2^ Tianjin Union Medical Center, Tianjin, China; ^3^ Medical Genetic Institute of Henan Province, Henan Provincial People’s Hospital, People’s Hospital of Zhengzhou University, Zhengzhou, Henan, China; ^4^ Department of Clinical Laboratory, the Fifth Central Hospital of Tianjin, Tianjin, China; ^5^ Jining Tumor Hospital, Jining No.1 People's Hospital North Campus, Shandong, China; ^6^ Traditional Chinese Medical Hospital of Changle, Shandong, China

**Keywords:** solid tumors, NRF2, prognosis, meta-analysis

## Abstract

Nuclear factor E2-related factor 2 (NRF2), a transcription factor, is known as a potential therapeutic target of solid tumor for that it is a master regulator of the injury and inflammation response, including controlling antioxidant cell progress. Recent studies showed that NRF2 played significant roles in tumorigenesis and tumor progression, however no association and relationship between NRF2 expression and different clinical manifestation of solid tumor had been accurately evaluated. The present meta-analysis picked up 17 suitable articles from EMBASE, PubMed, and ISI Web of Science databases, including 2238 patients. Combined with results of hazard ratios (HRs) and 95% confidence intervals (CIs), we concluded that a higher expression of NRF2 would have worse impact on overall survival (HR = 2.29, 95% CI 1.80–2.91, *P* < 0.05) and disease-free survival (HR = 2.34, 95% CI 1.36–4.00, *P* < 0.05) by a random-effect model. Moreover, further results were positively correlated to the clinical diagnosis, curative effect observation and prognosis, including tumor differentiation, lymph node metastasis, distant metastasis and clinical stage. Consequently, our data shown that NRF2 is a potential poor prognostic factor in a variety of solid tumors.

## INTRODUCTION

Oxidative stress played important roles in carcinogenesis [1]. Reactive oxygen species (ROS) damaged nucleotide, protein and lipids, which are generated by ionizing radiation [2], chronic inflammatory [3] or environmental agents [4, 5], then result in cell carcinogenesis. The intracellular ROS stimulated MAPK/PAK signal pathways [6], activated the downstream transcription factor (NRF2, AP1, NF-κB, and HIF-1α) [7] and consequently initiated downstream factors to degrade ROS and improved the cell survival.

Among the oxidative stress responsive transcription factors, NRF2 aroused extensive concern for its important role in cancer. NRF2 is a basic region-leucine zipper type transcription factor, combined with Keap1 (kelch like ECH associated protein 1) in basal stress condition and degraded by ubiquitin system through Keap1 at the same time [8]. In oxidative stress condition, the specific cysteines of Keap1 was oxidized or modified by ROS, which altered the conformation of Keap1 and derestricted NRF2 to activate its downstream anti-oxidative genes [9–11].

NRF2 is a double-edged sword in carcinogenesis. On the one hand, NRF2 degraded intracellular carcinogens by activating downstream genes to prevent carcinogenesis [12]. On the other hand, chemotherapy drugs increased activation of NRF2, which can counteract the role of drug molecules and promote the cell survival, finally result in chemotherapy resistance. Immunohistochemical researches in non-small cell lung cancer [13] and epithelial ovarian cancer [14] demonstrated that high expression of NRF2 is relevant to platinum resistant. Additionally, NRF2 itself can increase cell survival under stressing, and gain-of-function mutations of NRF2 have been found in various type of cancer, which loss its binding capacity with Keap1 but retains transcriptional activity [15]. Therefore, NRF2 is accounted by some researchers an “oncogenic” [11].

The complex roles of NRF2 in carcinogenesis remained wide interest and controversy in cancer biologists. More and more researchers have realized the “paradox” of NRF2 [7, 11, 12, 16] however, there is no comprehensive and quantitative analysis against the roles of NRF2 in carcinogenesis and cancer therapy. This meta-analysis aimed to evaluate the roles of NRF2 in carcinogenesis and prognosis from previous research data, and tried to provide a panoramic picture of NRF2 in cancer biology.

## RESULTS

### Study selection and characteristics description

**Figure 1:**
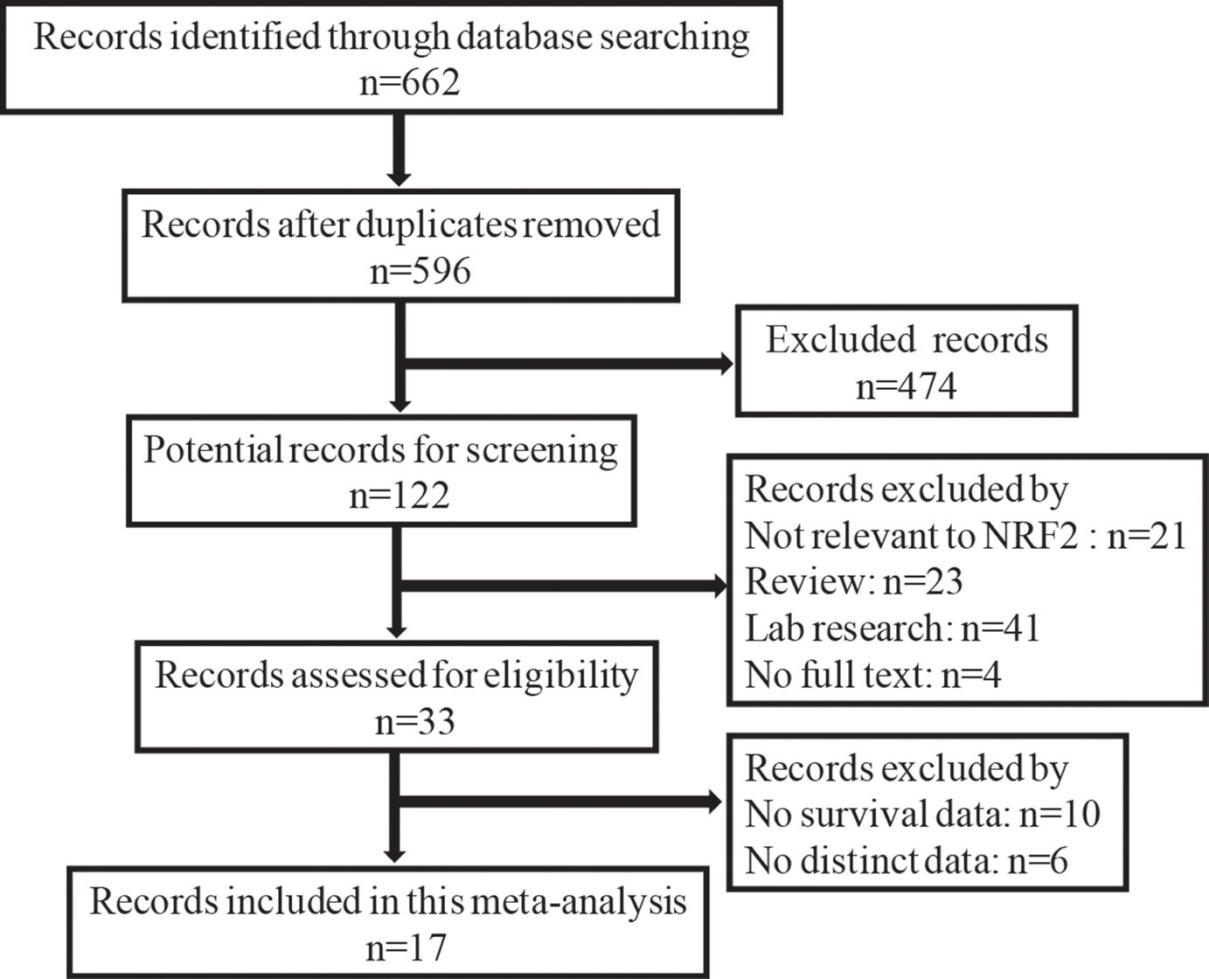
Flow diagram of the selection of eligible studies

The detailed study selection is shown as Figure 1. A total of 662 publications were identified in databases of EMBASE, PubMed, and ISI Web of Science. 645 of those were excluded, due to laboratory studies, articles (review), repetitive research, without full texts, detection method or studies irrelevant to the current analysis. 17 publications [13, 17–32], which includes 2238 patients, were included in this study. Each of the 17 eligible studies was assessed independently by two investigators according to the Newcastle–Ottawa Scale (NOS). Briefly, NOS are classified into the three dimensions of selection, comparability, and ascertainment of outcome. Each appraised study can be awarded a score ranged from 0 to 9. The score of 1–3, 4–6, 7–9 was defined as low, middle and high quality, respectively. Additionally, 11 of the studies obtained scores ≥ 7 in methodological assessment, indicating a high quality for the majority of these studies.

The participants in the studies covered kinds of countries and cancer types. The most focuses on China (47.06%) and Finland (23.53%), the rest are Japan (17.65%), America (5.89%) and Korea (5.89%). Main type of cancer among 2238 patients were lung cancer (*n* = 762), ovarian cancer (*n* = 208), gastric cancer (*n* = 175). Moreover, the median patient age ranged from 50.0 to 60.0 years. Additionally, >10% positive tumor cells and scores ≥ 1 were the most appropriate cut-off values for overall survival, > 10% positive tumor cells was a more suitable cut-off value for disease-free survival simultaneously. The detailed characteristics are listed in Table 1.

**Table 1:** Main characteristics of studies exploring the relationship between Nrf2 expression and tumor prognosis

Author	Year	Region	Cancer Type	Stage / Grade	No. of Patients	Age Median (Range)	Follow-up Time Median (range)	Detection Method	Cut-off	Outcomes	NOS Score
Onodera Y [17]	2014	Japan	BRCA	I–III	106	57 (31–81)	103 m (3–175)	IHC(sc-13032X)	≥ 10%	OS,DFS	7
Wang J [18]	2010	China	GBC	I–V	59	60	28.7 m (±14.4)	IHC(sc-722)	≥ 25%	OS	6
Kawasaki Y [19]	2015	Japan	GC	I–III	175	66 (31–84)	NR	IHC(sc-365949)	≥ 100%	OS	8
Zhao M [20]	2015	China	Gliomas	I–IV	75	50	5 y	IHC(Abcam)	> 10%	OS, DFS	6
Ji XJ [21]	2013	China	Glioblastoma	0–3	49	52 (27–82)	3 y	IHC(Abcam)	scores > 5	OS, PFS	5
Zhang M [22]	2015	China	HCC	0–3	80	60	16.6 m	IHC( Santa Cruz)	scores > 2	OS, DFS	6
Merikallio H [23]	2012	Finland	LC	NR	289	NR	NR	IHC( Santa Cruz)	≥ 5%	OS	8
Yang H [24]	2011	China	NSCLC	IIIB or IV	60	NR	NR	IHC(Beijing Bio)	≥ 50%	OS, PFS	6
Solis LM [13]	2010	USA	NSCLC	I-IV	304	66	5 y	IHC( Santa Cruz)	score > 0	OS, RFS	8
Inoue D [25]	2012	Japan	NSCLC	I-III	109	65.6 (23–82)	1626 d (17–3366)	IHC(sc-13032X)	≥10%	OS	7
Hintsala HR [26]	2016	Finland	Melanoma	I–V	121	70	NR	IHC(Santa Cruz)	NR	OS	7
Huang CF [29]	2013	China	OSCC	NR	43	NR	24 m (12–43)	IHC(Burlingame)	NR	OS, DFS	5
Zhang J [30]	2016	China	Osteosarcoma	NR	102	14	NR	IHC(Santa Cruz)	scores ≥ 3	OS	7
Liew PL [28]	2015	China	OC	I–IV	108	50	NR	IHC(NR)	≥ 50%	OS, DFS	7
Soini Y [31]	2014	Finland	PC	I–IV	103	NR	NR	IHC(Santa Cruz)	≥ 5%	OS	7
Raatikainen S [32]	2014	Finland	Prostate	NR	240	63	11.7 m (3.3–25.8)	IHC(Santa Cruz)	≥ 50%	OS, DFS	8
Cho HY [27]	2017	Korea	OC	1–3	100	55	55.3 m	IHC(Santa Cruz)	scores ≥ 1	OS, DFS	7

GC: Gastric Cancers; CRC: Colorectal Cancers; NPC: Nasopharyngeal Carcinoma; NSCLC: Non-Small Cell Lung Cancers; OC: Ovarian Cancers; OSCC: Oral Squamous Cell Carcinoma; HCC: Hepatocellular Carcinoma; SSCC: Sinonasal Squamous Cell Carcinoma; BRCA: Breast Cancer; GBC: Gallbladder Cancer ; Pancreatic adenocarcinoma: PC; lung cancer : LC; NR: Not Reported; y: year; m: month; d: day; OS: Overall Survival; DFS: Disease-Free Survival.

### NRF2 and overall survival

**Figure 2:**
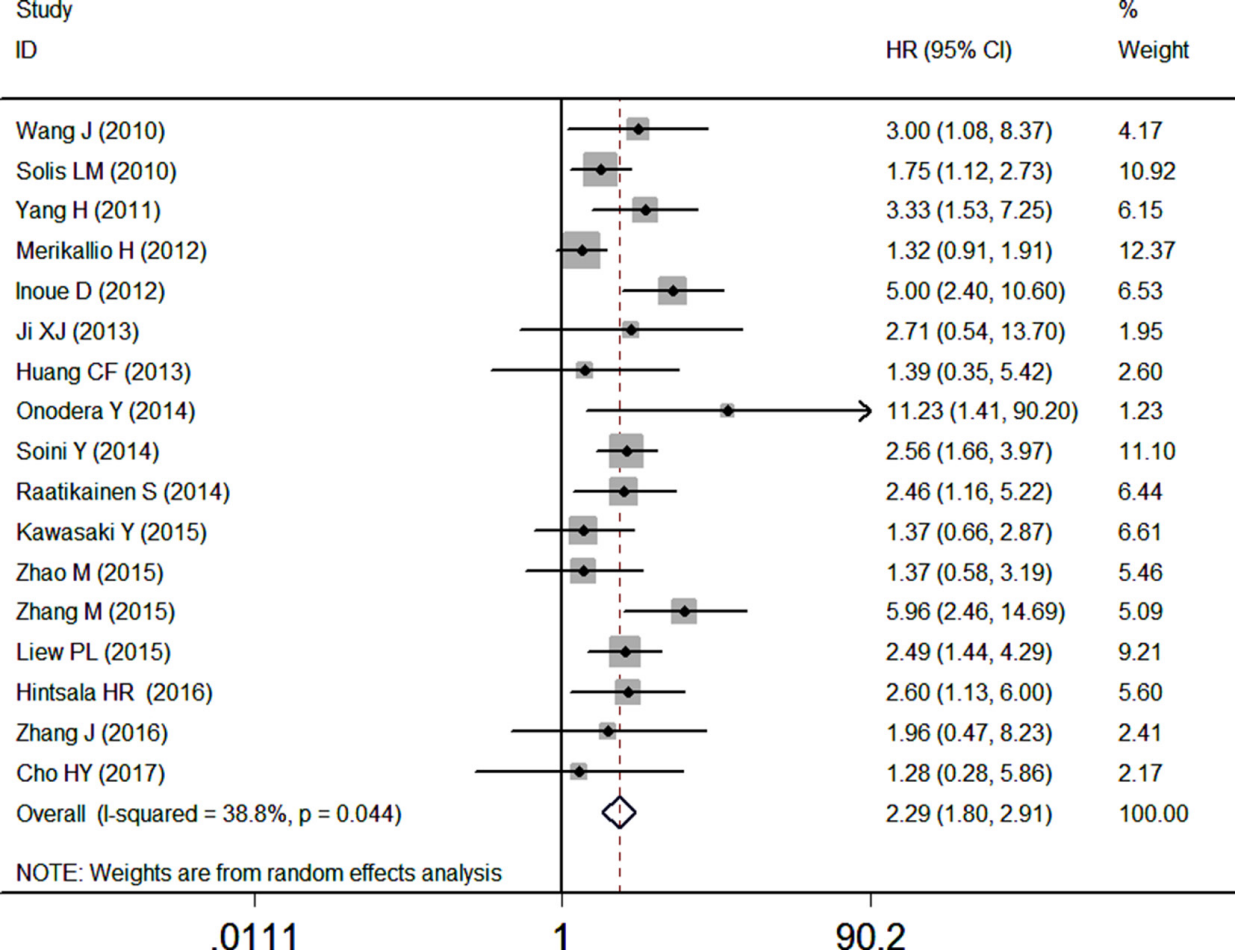
Forest plot describing the association between over-expressed NRF2 and OS

17 studies with data from 2238 patients were available to evaluate the effect of NRF2 expression on OS. The pooled HR revealed that over-expressed NRF2 was significantly associated with poor OS in multivariate analysis (HR: 2.29, 95% CI: 1.80–2.91, *P* < 0.05, Figure 2). Low significant heterogeneity (I^2^ = 38.8%) was observed when using a random-effects model to analyze the pooled HR of the OSs. This reveals excessive NRF2 expression had a poor prognosis in cancer patients (Figure 2).

### NRF2 and disease-free survival

**Figure 3:**
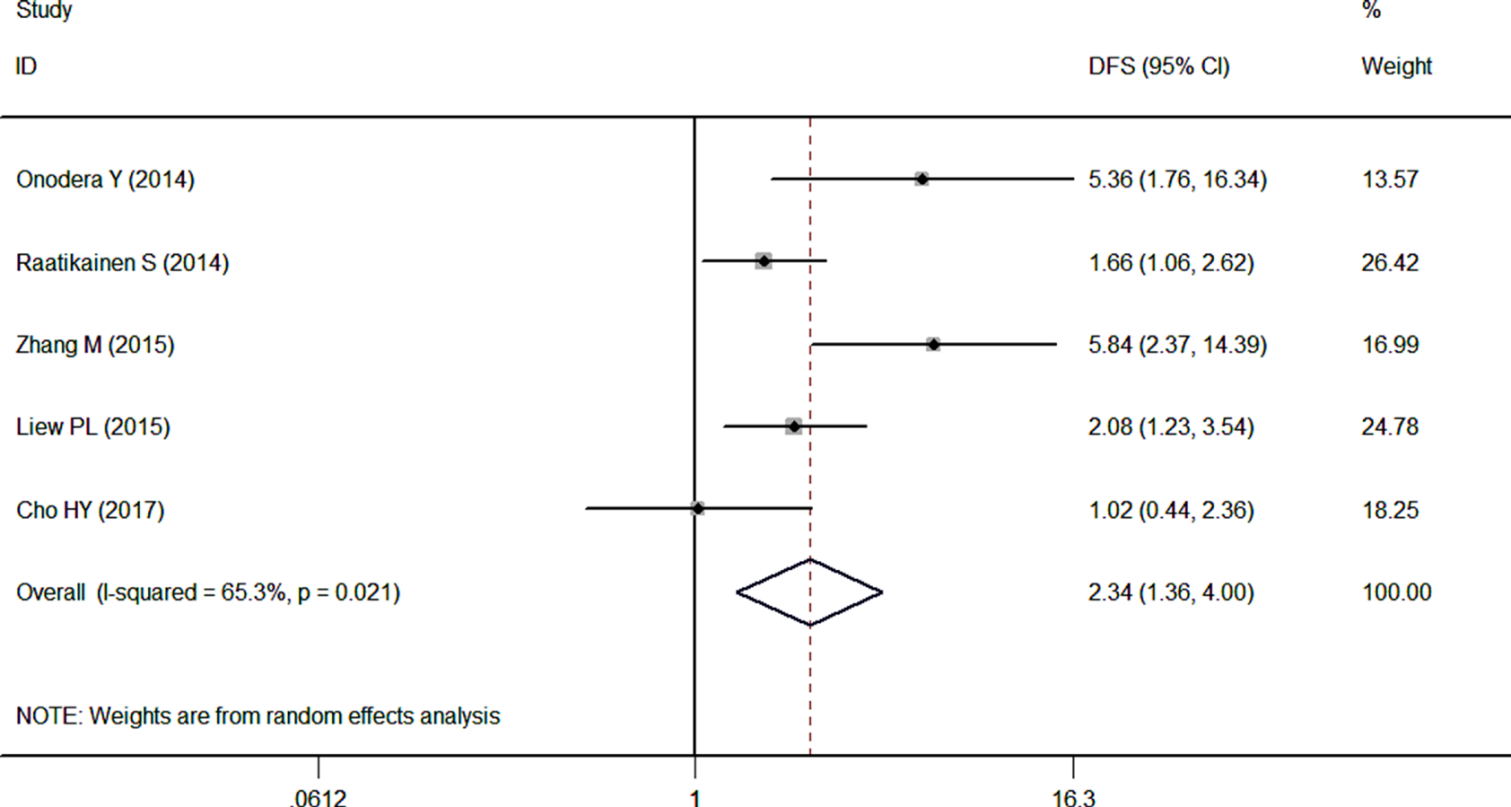
Forest plot describing the association between over-expressed NRF2 and DFS

Only 5 articles reported the relevance between the NRF2 expression and DFS, the pooled HR was 2.34 (95% CI: 1.36–4.00, *P* < 0.05) with high heterogeneity (I^2^ = 65.3%) by a random-effects model. The results in Figure 3 indicated that the high expression of NRF2 is worse than the control group (Figure 3).

### NRF2 and slinicopathological parameters

The clinical and pathological parameters collected from the eligible studies were presented in Table 2. Meanwhile, pooled results of the correlations were identified between the high expression of NRF2 and clinicopathological features of patients with solid tumors. We found that the over-expressed NRF2 was positively associated with tumor differentiation (OR = 2.72, 95% CI: 1.30–5.70), lymph node metastasis (OR = 2.07, 95% CI: 1.13–3.78), distant metastasis (OR = 8.21, 95% CI: 1.57–43.00) and clinical stage (OR = 3.37, 95%CI: 1.98–5.73) with statistical significance according to the multivariate analysis. However, NRF2 was not relevant to other clinicopathological features such as the gender (OR = 1.28, 95% CI: 0.73–2.24) and tumor size status (OR = 2.11, 95% CI: 0.88–5.03) (Table 3).

**Table 2:** Summarized data of clinical and pathological parameters from the eligible studies

First author Nrf2	Gender				Tumor differentiation				Tumor size				Lymph node metastasis				Distant metastasis				Clinical stage			
	Male		Female		Poor+Moderate/ undifferentiated		Well/ differentiated		T3–4		T1–2		Yes		No		Yes		No		III–IV		I–II	
	+	−	+	−	+	−	+	−	+	−	+	−	+	−	+	−	+	−	+	−	+	−	+	−
Onodera Y [17]	NA	NA	NA	NA	42	44	5	15		29	16	30	27	24	20	35	NA	NA	NA	NA	14	10	33	49
Wang J [18]	13	5	32	9	44	10	1	4	NA	NA	NA	NA	27	0	18	14	18	0	27	14	21	6	6	8
Kawasaki Y [19]	65	51	43	16	74	30	34	37	89	36	19	31	74	35	34	32	NA	NA	NA	NA	68	27	40	40
Zhao M [20]	30	15	16	14	NA	NA	NA	NA	NA	NA	NA	NA	NA	NA	NA	NA	NA	NA	NA	NA	30	6	16	23
Zhang M [22]	34	10	14	7	28	2	20	15	27	4	21	13	NA	NA	NA	NA	29	1	19	16	NA	NA	NA	NA
Yang H [24]	26	14	8	12	17	9	8	8	NA	NA	NA	NA	NA	NA	NA	NA	NA	NA	NA	NA	28	14	6	12
Inoue D [25]	31	47	6	25	10	23	27	49	21	48	16	24	14	21	23	51	NA	NA	NA	NA	NA	NA	NA	NA
Zhang J [30]	37	11	42	12	NA	NA	NA	NA	NA	NA	NA	NA	NA	NA	NA	NA	40	9	32	21	NA	NA	NA	NA

**Table 3:** Meta-analysis results of the associations of increased Nrf2 expression with clinicopathological parameters

Clinicopathological parameter	Ref	Overall OR (95%CI)	*P*-value	Heterogeneity test (I^2^, *P*-value)
Gender (male vs female)	[18–20], [22], [24, 25], [30]	1.28 (0.73–2.24)	0.392	(56.2%, 0.033)
Differentiation (poor VS well)	[17–20], [24, 25]	2.72 (1.30–5.70)	0.008	(60.8%, 0.026)
Tumor size (T3–4 vs T1–2)	[17], [19], [22], [25]	2.11 (0.88–5.03)	0.094	(75.9%, 0.006)
Lymph node metastasis (yes vs no)	[17–19] ,[25]	2.07 (1.13–3.78)	0.018	(42.6%, 0.156)
Distant metastasis (yes vs no)	[18], [22], [30]	8.21 (1.57–43.00)	0.013	(57.8%, 0.094)
Clinical stage (III-IV vs I-II)	[17–20], [24]	3.37 (1.98–5.73)	0.000	(24.2%, 0.260)

### Assessment of heterogeneity and sensitivity

**Figure 4:**
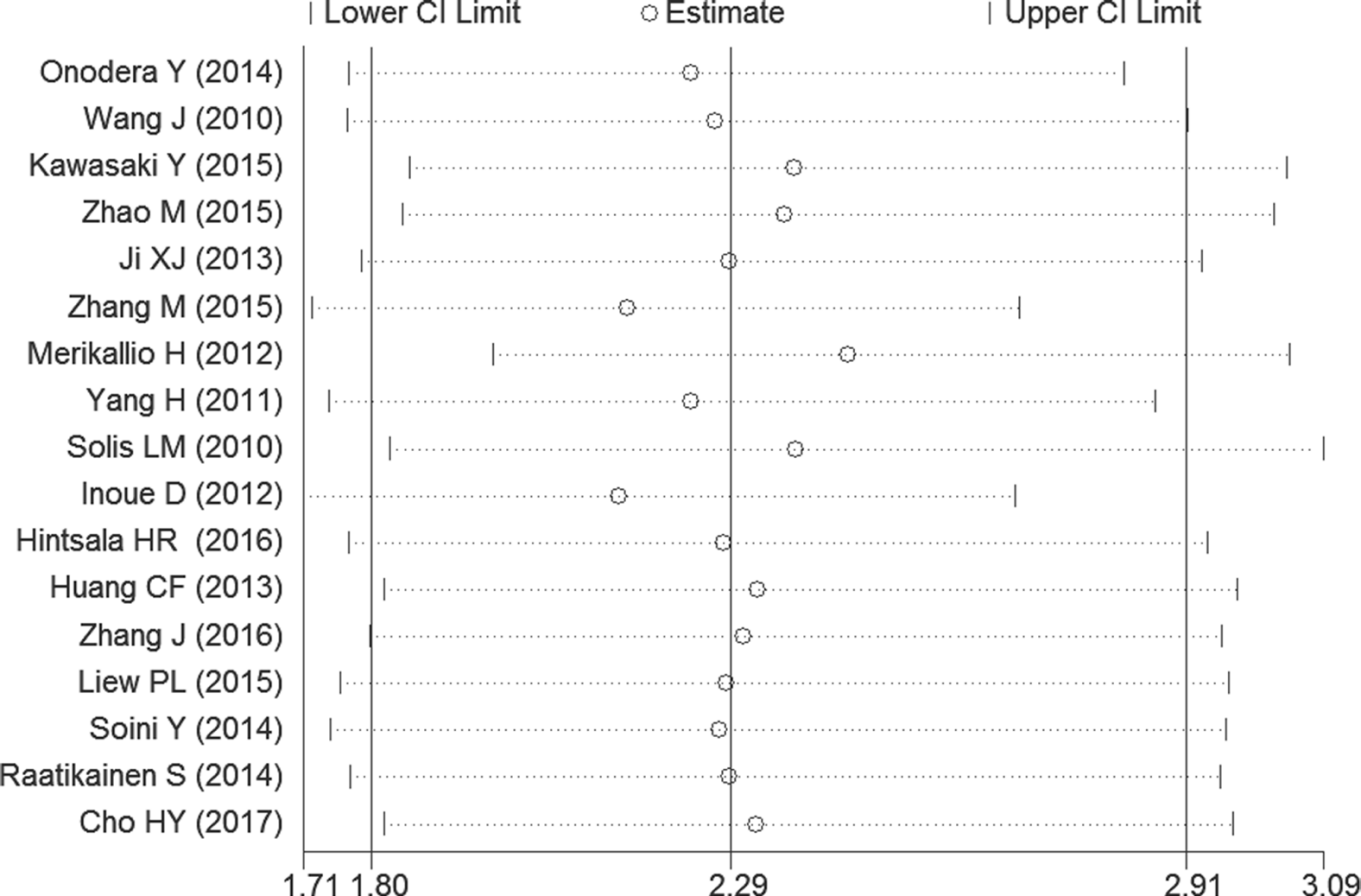
Sensitivity analysis of the OS in the meta-analysis

There was low heterogeneity (I^2^ > 30%) between studies in OS analyses. So the random-effect model was therefore adopted in these studies. By successively omitting each study from the aggregated survival meta-analyses, a sensitivity analysis was performed to evaluate the influence of each individual study on the pooled HR (Figure 4). The results revealed that the pooled estimates of the effect of over-expressed NRF2 on the OS of patients with solid tumors did not vary substantially with the exclusion of any individual study, which implies that the results of this meta-analysis are stable.

### Publication bias

**Figure 5:**
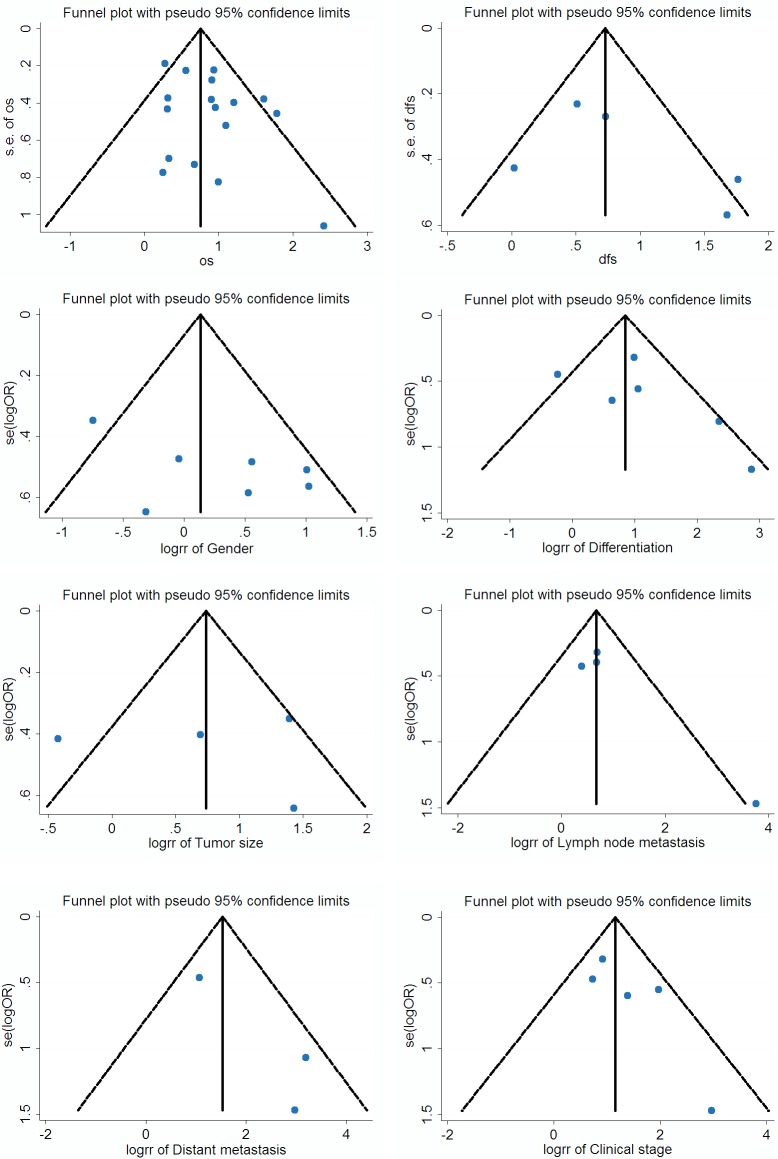
Funnel plot for the assessment of potential publication bias regarding OS, DFS and clinicopathological features of patients in the meta-analysis

We constructed Begg’s funnel plot with pseudo 95% confidence limits tests to evaluate the publication bias risk in these applicable studies. The shapes of the funnel plots for OS, DFS and clinicopathological features of patients were almost symmetrical, indicated that there is no statistically significant difference, therefore no significant publication bias (Figure 5). Thus, in these incorporated papers, it was found that there was no evidence of significant publication bias after assessing and the results of this meta-analysis are reliable.

## DISCUSSION

Our meta-analysis demonstrated that the excessive expression of NRF2 was related to poor OS (HR = 2.29, 95% CI: 1.80–2.91, *P* < 0.05) and DFS (HR = 2.34, 95% CI 1.36–4.00, *P* < 0.05) in various type of cancer patients. Also, the association between NRF2 expression and different clinicopathological parameters was consistent, which indicated general roles of NRF2 in cancer prognosis. NRF2-Keap1 pathway played essential roles in the response of oxidative stress, but exhibited “double-edged” effect in carcinogenesis. This meta-analysis help us to re-understand and re-discovery the roles of NRF2 in tumorigenesis, and figured out the relevance between NRF2 expression and patients’ survival. The present meta-analysis exhibited no publication bias for OS according to the funnel plot and Begg’s test. Thus, the results in our study has a high degree of credibility.

Given the difference of tumor types and population, the sensitivity of OS in most articles reflected the relevance between NRF2 level and carcinogenesis is stable, and the prognosis value of NRF2 is universal. Nevertheless, there are several limitations on this article worthy of our attention. A potential limitation is the high heterogeneity across diverse publications for DFS and different clinicopathological parameters. The number of samples was small, which is not sufficient to detect a remarkably difference between them.

Another limitation of our meta-analysis is lack of stratified analysis for different tumor subtype. Although there are many studies on NRF2 in various type of cancer, little of them focused on subtype of specific cancer. For breast cancer, though, the sub-classification of breast cancer was also classified according to ER-positive and negative [17], the lack of ductal carcinoma, adenocarcinoma such morphological classification of discussion, and this degree of malignancy for breast cancer and NRF2 correlation also has important reference value. For lung cancer, the study of NRF2 mainly focused on non-small cell lung cancer [13, 24], but less focused on small cell lung cancer subtypes [23, 25]. For other cancers, many of them have no stratified analysis for different tumor subtype.

A possible mechanism for the poor prognosis with excessive NRF2 expression can be inferred from the existing studies of NRF2: First, NRF2 promotes tumorgenicity itself. A research in 2011 indicate that oncogenes like KRAS, MYC and BRAF can promote the expression of NRF2 to degrade ROS to make a more reduced intercellular environment, therefore, protects cancer cell from oxidative stress and promote cell proliferation and tumorigenecity [33]. Second, NRF2 mediated various chemotherapy drugs resistance, which is reported by the researches in lung cancer [13] and ovarian cancer [14] by studying in cell lines and animal models [34–36].

For the “paradox” of NRF2’ role in tumorigenesis, some researchers thought that NRF2 may act diversely roles in different expression level [12] and different stage of tumorigenesis [16]: It can prevent tumorigenesis in premalignant cell or early stage malignant cell, but promote tumorigenesis and chemotherapy resistance in malignant cell, which is like the role of TGF-β in tumorigenesis [37]. Further stratified analysis of the roles of NRF2 in different stage of cancer patients can be performed to verify this hypothesis in the future. Furthermore, quantitative biological technique can be also utilized to quantify the level of NRF2 in the whole process of tumorigenesis to give more instructive advice for the prognosis of different stage of cancer. Whether meta-analysis can be used for different periods of cancer patients with their survival and NRF2 expression level analysis, to verify or deny this view? Such analysis will arouse the interest of the majority of researchers.

## MATERIALS AND METHODS

### Search strategy and selection criteria

We systematically select the appropriate articles from the EMBASE, PubMed, and ISI Web of science databases using the following keywords in all possible combinations: “NRF2/Nuclear factor E2-related factor 2, cancer/tumor, prognosis/prognostic/ survival”. As of March 31, 2017, all publications on NRF2 are eligible for inclusion except for the following: (1) reviews, letters, editorials, and expert opinion, case reports; (2) overlapping articles or irrelevant article; (3) articles that cannot be extracted the original data; and (4) insufficient data for estimating the odds ratio (OR) or hazard ratio (HR) and 95% confidence interval (CI) [38]. The articles we selected are subject to the following conditions: (1) the expression of NRF2 is detected by immunohistochemistry in patients; (2) associations of NRF2 expression with overall survival (OS) or disease-free survival (DFS); (3) pathological diagnosis of different tumor types or clinicopathological features were described; and (4) When the same author reports the same crowd, the most complete result is hired.

### Data extraction

Two investigators (Chunze Zhang and Litao Qin) independently used a standardized data-extract form, and collecting information as follows: first author’s name, publication date, the patient’s region, type of cancer, number of patients, NRF2 detection method, antibody source, number of patients with NRF2-positive, follow-up times, cut-off values, and survival data (Table 1) . Any disagreements were adjudicated by discussion until a consensus was reached [39].

### Statistical method of meta-analyses

This meta-analysis was performed using Stata 12.0 (Stata Corporation, College 216 Station, TX, USA) software. The results of multivariate analysis were based on the Cox proportional hazard model. Pooled estimates of HRs and their 95% CIs were used to estimate the association between NRF2 expression and patients’ survival. The chisquared test (Cochrane’ s *Q* test) and I-squared statistical test were used to analyze the heterogeneity between studies. When the result of a *Q*-test (*I*^2^ > 30% or *P* < 0.05) indicated heterogeneity, the random-effects model was used for the meta-analysis [40]. HR with its value over 1.0 indicated poor prognosis patients with increased NRF2 expression. Beyond that, we estimated data by sampling variation for the calculation of ORs and 95% confidence interval (CI) [41], which was used to evaluate the correlation between NRF2 expression and the risk of clinical patients’ diagnosis, such as clinical stage, lymph node metastasis and distant metastasis. Moreover, Begg’s funnel plot aimed to examine the potential risk of publication bias [42, 43]. *P* ≤ 0.05 was considered to be statistically significant in this analysis.
